# Long-Term Persistence of Anti-Poliovirus Antibody Titers After Two-Dose Booster Immunization with Conventional Inactivated Poliovirus Vaccine Among Japanese Adults: 10-Year Observational Study

**DOI:** 10.3390/vaccines13050447

**Published:** 2025-04-23

**Authors:** Shinji Fukushima, Takashi Nakano, Minetaro Arita, Hiroyuki Shimizu, Atsuo Hamada

**Affiliations:** 1 Travellers’ Medical Center, Tokyo Medical University Hospital, 6-7-1 Nishishinjuku, Shinjuku City 160-0023, Tokyo, Japan; a-hamada@tokyo-med.ac.jp; 2 Department of Pediatrics, Kawasaki Medical School, 577 Matsushima, Kurashiki 701-0192, Okayama, Japan; nakano@med.kawasaki-m.ac.jp; 3 Department of Virology II, National Institute of Infectious Diseases, 4-7-1 Gakuen, Musashimurayama-shi 208-0011, Tokyo, Japan; minetaro@niid.go.jp (M.A.); hshimizu@niid.go.jp (H.S.)

**Keywords:** long-term immunity, conventional inactivated poliovirus vaccine, booster immunization, immunogenicity

## Abstract

**Background/Objectives**: Most Japanese adults received two doses of the oral polio vaccine (OPV) during childhood as part of the national immunization program. However, these two doses are considered suboptimal by global standards. The long-term persistence of anti-poliovirus antibodies after booster doses with the conventional inactivated poliovirus vaccine (cIPV) in Japanese adults remains unclear. This study was performed to evaluate long-term immunogenicity over a 10-year period following two cIPV booster vaccinations. **Methods**: Ten out of sixty-one adult participants in a short-term study were enrolled to assess the long-term immunogenicity of the booster vaccination. They underwent blood sampling at 3, 5, and 10 years after cIPV vaccination. **Results**: The results indicate that, even 10 years after the booster vaccination, antibodies against poliovirus types 1 and 2 remained at high levels, exceeding the detection limits of neutralization tests. However, some participants showed decreased antibody levels against poliovirus type 3. **Conclusions**: This study showed that cIPV boosters provided long-lasting protective immunity against poliovirus types 1 and 2 in adults who were vaccinated with OPV. These findings are valuable in assessing the need for IPV booster vaccinations in adults.

## 1. Introduction

When the World Health Organization launched its global polio eradication program in 1988, the estimated number of polio cases caused by wild poliovirus (WPV) was 350,000. In 2023, Afghanistan and Pakistan reported 12 cases caused by WPV type 1 [1,2]. Wild poliovirus types 2 and 3 have been eradicated; however, 529 cases of circulating vaccine-derived poliovirus (cVDPV) were confirmed in 2023. Of these, 395 were cVDPV type 2 (cVDPV2) and 134 were cVDPV type 1 (VDPV1) [3]. Although the polio eradication plan is ongoing, polio continues to be classified as a Public Health Emergency of International Concern [3]. A case of acute flaccid myelitis associated with cVDPV2 was identified in the United States, and cVDPV2 was detected in environmental sewage samples in the United Kingdom, Canada, and Israel [4,5,6,7,8].

Preventing the spread of WPV and controlling polio outbreaks caused by cVDPV are both essential elements of global polio eradication strategies. The Polio Eradication Strategy 2022–2026 presents a comprehensive action plan that positions the Global Polio Eradication Initiative to achieve its goal of eradicating polio. This strategy outlines two primary goals: first, to permanently halt all poliovirus transmission in WPV-endemic countries, specifically Afghanistan and Pakistan, and second, to stop cVDPV transmission and prevent outbreaks in non-endemic countries [9]. The plan encourages countries to strengthen routine childhood immunization programs, introduce inactivated poliovirus vaccine (IPV) into routine immunization schedules, and gradually transition from the trivalent oral poliovirus vaccine (tOPV) to the bivalent OPV [9].

In Japan, routine childhood immunization programs include vaccines against polio, tuberculosis, and measles/rubella [10,11]. As of March 2024, all infants had received four doses of inactivated poliovirus-containing vaccine, either alone or in combination with the diphtheria, tetanus, and pertussis vaccine. The vaccination rate for polio in Japan is high, and the disease is considered under control [12]. However, because polio remains endemic in some regions globally, careful monitoring of WPV and cVDPV importation and transmission is necessary. Most Japanese individuals aged ≥ 12 years in 2024 received two doses of tOPV (Sabin strains) in childhood as part of the national immunization program, although two doses of tOPV are considered suboptimal internationally [13].

In our previous short-term study, we administered two-dose catch-up vaccination with conventional IPV (cIPV) to Japanese adults and measured antibody titers before and after vaccination (1 month after the first booster vaccination and 1 month after the second booster vaccination) [14]. However, data on the long-term persistence of antibody titers conferred by the two-dose booster vaccination of cIPV among adults are unclear.

This study was performed to evaluate long-term immunogenicity over a 10-year period following the previous short-term study.

## 2. Materials and Methods

### 2.1. Study Design, Participants and Study Procedures

This long-term serological follow-up study was an observational study conducted at the Tokyo Medical University Hospital from 25 February 2015 to 30 September 2024.

We contacted Japanese adults who participated in the previous short-term study by email approximately 3 years after the two-dose booster was administered. Of the 61 participants in the prior short-term study, 10 were successfully contacted. These 10 participants were included in this long-term study. They visited Tokyo Medical University Hospital and underwent blood sampling at 3, 5 and 10 years after receiving the two-dose booster vaccination.

### 2.2. Laboratory Testing

To evaluate poliovirus neutralizing antibody titers, 6 mL blood samples were collected in dry, sterile, capped plastic tubes. After allowing the blood to clot, the serum was isolated via centrifugation at 1500× *g* for 10 min. The serum samples were stored at temperatures of ≤−20 °C until the measurement of neutralization antibody titers.

Neutralization antibody titers were measured at the National Institute of Infectious Diseases (Tokyo, Japan). For the 3-year blood samples, immunogenicity was assessed by measuring neutralizing antibody titers against type 1, 2, and 3 polioviruses in HEp-2 cells using a microneutralization assay. For the 5- and 10-year blood samples, immunogenicity was assessed using a pseudovirus poliovirus neutralization test with type 1, 2, and 3 poliovirus pseudoviruses [15].

The neutralization antibody titer required for protection against poliovirus was assumed to be 1:8 (serum dilution) or higher [16]. Titers below 1:4 were assigned a value of 1:2. Titers above 1:512 were capped at 1:512.

### 2.3. Statistical Analysis

No hypothesis testing was conducted in this study; descriptive statistics were used to summarize the data. Seropositivity rates were calculated to assess the immunogenicity of poliovirus vaccines. The seropositivity rates and the geometric mean titers (GMT) were determined pre-vaccination, post-vaccination after the two doses of cIPV, and at the 3-, 5-, and 10-year follow-ups. Seropositivity was defined as the percentage of participants with neutralization antibody titers of ≥1:8.

All the statistical analyses were performed using EZR version 1.61 (Saitama Medical Center, Jichi Medical University, Saitama, Japan) [17], which is a modified version of R Commander (version 2.8-0) designed to include statistical functions commonly used in biostatistics.

### 2.4. Ethics

This study was approved by the Institutional Review Board of Tokyo Medical University, and was conducted following the ethical principles laid out in the Declaration of Helsinki. All participants provided written informed consent before participating in this study.

## 3. Results

### 3.1. Baseline Characteristics

The ten participants comprised four men and six women with a median age of 35.5 years (range: 28–56). Five participants had maternal and child health handbooks documenting that they had received two doses of OPV.

### 3.2. Poliovirus Antibody Titers Against Poliovirus

The seropositivity rates (defined as neutralizing antibody titers ≥ 1:8) were as follows: at 3 years—Sabin 1 (100%), Sabin 2 (100%), Sabin 3 (100%), Mahoney (100%), MEF-1 (100%), and Saukett (100%); at 5 years—Sabin 1 (100%), Sabin 2 (100%), Sabin 3 (80%), Mahoney (100%), MEF-1 (100%), and Saukett (90%); and at 10 years—Sabin 1 (100%), Sa-bin 2 (100%), Sabin 3 (80%), Mahoney (100%), MEF-1 (100%), and Saukett (90%) (Table 1).

The GMTs of the anti-poliovirus antibodies are shown in Table 2. For the Sabin strains (types 1, 2, and 3), the GMTs decreased from 512, 512, and 222.9 following the two-dose booster vaccination to 477.7, 415.9, and 147.0 after 3 years; to 274.4, 294.1, and 73.5 after 5 years; and to 238.9, 294.1, and 59.7 after 10 years, respectively. For the virulent poliovirus strains (type 1: Mahoney strain; type 2: MEF-1 strain; and type 3: Saukett strain), the GMTs decreased from 512, 512, and 256 following the two-dose booster vaccination to 362, 388, and 111.4 after 3 years; to 238.9, 337.8, and 97 after 5 years; and to 168.9, 362, and 59.7 after 10 years, respectively.

## 4. Discussion

Our prior short-term study showed that some Japanese adults maintained poliovirus neutralization antibody titers at protective levels even before receiving cIPV vaccination. This result suggests that tOPV provided effective long-term protection against poliovirus. However, neutralization antibody titers against the Sabin 3 and Saukett strains were relatively low at baseline [14]. This trend is consistent with data previously reported by the National Institute of Infectious Diseases [18,19].

Japanese adults are considered incompletely vaccinated against poliovirus by global standards because they received only two doses of tOPV in childhood [13]. For this reason, it is recommended that Japanese adults traveling to polio-endemic or high-risk areas receive a booster dose of the poliovirus vaccine. These incompletely vaccinated adults should receive one or two booster doses of cIPV [13,20,21]. Our prior study demonstrated that antibody titers increased significantly after booster vaccination, reaching protective levels against all poliovirus strains [14].

In this long-term study, we evaluated the course of immunogenicity in Japanese adults who received a booster dose of cIPV. Antibody titers for all strains (WPV and Sabin strains) decreased over time, with measurements taken at 3, 5, and 10 years. Ten years after receiving the booster vaccinations, all participants remained positive for antibodies against poliovirus types 1 and 2, but some had become negative for poliovirus type 3.

The low prevalence of antibodies against poliovirus type 3 in adults aligns with data from other countries [22,23,24,25].

Antibody titers for poliovirus type 3 may decline more rapidly compared to types 1 and 2. Among the participants in our long-term study, one individual had a history of receiving two doses of OPV (oral poliovirus vaccine), but at the time of their participation in the short-term study, they were negative for antibody titers against Sabin 2, Sabin 3, Mahoney, MEF-1, and Saukett. Additionally, three other participants were negative for antibody titers against Sabin 3 and Saukett.

The antibody titers against Sabin 3 and Saukett, which were negative at the time of participation in the short-term study, were inferred to respond poorly even after booster vaccination of cIPV, declining over a long period and eventually becoming negative. On the other hand, the antibody titers for types 1 and 2 increased significantly to 512-fold following additional cIPV vaccinations, and did not become negative despite decreasing over time.

Since March 2022, outbreaks of cVDPV3 have been reported in French Guiana (a French territory in South America) and Guinea in the African region. Three environmental samples tested positive for cVDPV3 in French Guiana, and three cases of paralysis have been reported in Guinea [3]. Therefore, travelers to countries where cVDPV3 is endemic should be vigilant even if they have previously received a booster dose of cIPV

This study is limited by its single-center design and small sample size, making generalization difficult. Additionally, the methods for measuring poliovirus antibodies differed after 5 and 10 years compared with the previous study because of the strict biorisk management protocols currently being implemented for poliovirus infectious materials. Despite these limitations, it is suggested that antibody titers against poliovirus types 1 and 2 may be maintained in the long term if adults previously vaccinated with tOPV receive a booster with cIPV. Moreover, it was revealed that even within the small sample size, there were some cases where antibody titers against poliovirus type 3 diminished.

## 5. Conclusions

This study showed that cIPV boosters provided long-lasting protective immunity against poliovirus types 1 and 2 in adults who were vaccinated with incomplete doses of OPV but whose antibody titers against poliovirus type 3 declined. These findings suggest that booster vaccinations should be appropriately considered prior to travel, especially for adults traveling to countries where poliovirus type 3 is endemic.

## Figures and Tables

**Table 1:** Seropositivity rates against poliovirus strains.

		Prior Short-Term Study			Long-Term Study		
		Pre	Post 1	Post 2	3 Years	5 Years	10 Years
		% (95CI)	% (95%CI)	% (95%CI)	% (95%CI)	% (95%CI)	% (95%CI)
Sabin	Sabin 1	100 (69.2–100)	100 (69.2–100)	100 (69.2–100)	100 (69.2–100)	100 (69.2–100)	100 (69.2–100)
	Sabin 2	90 (55.5–99.7)	100 (69.2–100)	100 (69.2–100)	100 (69.2–100)	100 (69.2–100)	100 (69.2–100)
	Sabin 3	60 (26.2–87.8)	100 (69.2–100)	100 (69.2–100)	100 (69.2–100)	80 (94.1–100)	80 (94.1–100)
Virulent	Mahoney (Type 1)	90 (55.5–99.7)	100 (69.2–100)	100 (69.2–100)	100 (69.2–100)	100 (69.2–100)	100 (69.2–100)
	MEF-1 (Type 2)	90 (55.5–99.7)	100 (69.2–100)	100 (69.2–100)	100 (69.2–100)	100 (69.2–100)	100 (69.2–100)
	Saukett (Type 3)	60 (55.5–99.7)	100 (69.2–100)	100 (69.2–100)	100 (69.2–100)	90 (94.1–100)	90 (94.1–100)

Pre, pre-booster vaccination; Post 1, 1 month after first dose booster; Post 2, 1 month after second dose booster.

**Table 2:** GMTs against poliovirus strains.

		Prior Short-Term Study						Long-Term Study					
		Pre		Post 1		Post 2		3 Years		5 Years		10 Years	
		GMT	(95%CI)	GMT	(95%CI)	GMT	(95%CI)	GMT	(95%CI)	GMT	(95%CI)	GMT	(95%CI)
Sabin	Sabin 1	64.0	(25.1; 162.9)	512		512		477.7	(408.3; 558.5)	274.4	(190.1; 395.4)	238.9	(154.9; 369.0)
	Sabin 2	55.7	(23.4; 132.7)	512		512		415.9	(297.9; 580.8)	294.1	(186.6; 463.4)	294.1	(176.2; 490.9)
	Sabin 3	13.9	(3.7; 53.1)	222.9	(90.8; 548.3)	222.9	(90.8; 548.3)	147.0	(49.3; 437.5)	73.5	(16.5; 327.3)	59.7	(12.6; 283.8)
Virulent	Mahoney (Type 1)	22.6	(9.7; 53.0)	477.7	(408.3; 558.5)	512		362.0	(254.7; 514.0)	238.9	(126.2; 451.9)	168.9	(77.3; 369.0)
	MEF-1 (Type 2)	59.7	(22.5; 158.5)	512		512		388.0	(300.6; 501.2)	337.8	(222.3; 512.9)	362.0	(223.4; 586.1)
	Saukett (Type 3)	9.2	(3.3; 25.3)	256	(95.1; 690.2)	256	(97.7; 671.4)	111.4	(37.4; 331.9)	97.0	(31.5; 299.2)	59.7	(15.8; 225.9)

Pre, pre-booster vaccination; Post 1, 1 month after first dose booster; Post 2, 1 month after second dose booster. GMT, geometric mean antibody titer; CI, confidence interval.

## Data Availability

The dataset is available upon request.

## Abbreviations

WPV	wild poliovirus
OPV	oral poliovirus vaccine
tOPV	trivalent oral poliovirus vaccine
cVDPV	circulating vaccine-derived poliovirus
IPV	inactivated poliovirus vaccine
cIPV	conventional inactivated poliovirus vaccine
GMT	geometric mean titer
